# Systematic investigation of genetically determined plasma and urinary metabolites to discover potential interventional targets for colorectal cancer

**DOI:** 10.1093/jnci/djae089

**Published:** 2024-04-22

**Authors:** Jing Sun, Jianhui Zhao, Siyun Zhou, Xinxuan Li, Tengfei Li, Lijuan Wang, Shuai Yuan, Dong Chen, Philip J Law, Susanna C Larsson, Susan M Farrington, Richard S Houlston, Malcolm G Dunlop, Evropi Theodoratou, Xue Li

**Affiliations:** Department of Big Data in Health Science School of Public Health, and Center of Clinical Big Data and Analytics of The Second Affiliated Hospital, Zhejiang University School of Medicine, Hangzhou, Zhejiang, China; Department of Big Data in Health Science School of Public Health, and Center of Clinical Big Data and Analytics of The Second Affiliated Hospital, Zhejiang University School of Medicine, Hangzhou, Zhejiang, China; Department of Big Data in Health Science School of Public Health, and Center of Clinical Big Data and Analytics of The Second Affiliated Hospital, Zhejiang University School of Medicine, Hangzhou, Zhejiang, China; Department of Big Data in Health Science School of Public Health, and Center of Clinical Big Data and Analytics of The Second Affiliated Hospital, Zhejiang University School of Medicine, Hangzhou, Zhejiang, China; Department of Big Data in Health Science School of Public Health, and Center of Clinical Big Data and Analytics of The Second Affiliated Hospital, Zhejiang University School of Medicine, Hangzhou, Zhejiang, China; Centre for Global Health, Usher Institute, University of Edinburgh, Edinburgh, UK; Unit of Cardiovascular and Nutritional Epidemiology, Institute of Environmental Medicine, Karolinska Institutet, Stockholm, Sweden; Department of Colorectal Surgery, The First Affiliated Hospital, Zhejiang University School of Medicine, Hangzhou, Zhejiang, China; Division of Genetics and Epidemiology, The Institute of Cancer Research, London, UK; Unit of Cardiovascular and Nutritional Epidemiology, Institute of Environmental Medicine, Karolinska Institutet, Stockholm, Sweden; Unit of Medical Epidemiology, Department of Surgical Sciences, Uppsala University, Uppsala, Sweden; Cancer Research UK Edinburgh Centre, Medical Research Council Institute of Genetics and Cancer, University of Edinburgh, Edinburgh, UK; Division of Genetics and Epidemiology, The Institute of Cancer Research, London, UK; Cancer Research UK Edinburgh Centre, Medical Research Council Institute of Genetics and Cancer, University of Edinburgh, Edinburgh, UK; Colon Cancer Genetics Group, Institute of Genetics and Cancer, University of Edinburgh, Edinburgh, UK; Centre for Global Health, Usher Institute, University of Edinburgh, Edinburgh, UK; Colon Cancer Genetics Group, Institute of Genetics and Cancer, University of Edinburgh, Edinburgh, UK; Department of Big Data in Health Science School of Public Health, and Center of Clinical Big Data and Analytics of The Second Affiliated Hospital, Zhejiang University School of Medicine, Hangzhou, Zhejiang, China

## Abstract

**Background:**

We aimed to identify plasma and urinary metabolites related to colorectal cancer (CRC) risk and elucidate their mediator role in the associations between modifiable risk factors and CRC.

**Methods:**

Metabolite quantitative trait loci were derived from 2 published metabolomics genome-wide association studies, and summary-level data were extracted for 651 plasma metabolites and 208 urinary metabolites. Genetic associations with CRC were obtained from a large-scale genome-wide association study meta-analysis (100* *204 cases, 154* *587 controls) and the FinnGen cohort (4957 cases, 304* *197 controls). Mendelian randomization and colocalization analyses were performed to evaluate the causal roles of metabolites in CRC. Druggability evaluation was employed to prioritize potential therapeutic targets. Multivariable Mendelian randomization and mediation estimation were conducted to elucidate the mediating effects of metabolites on the associations between modifiable risk factors and CRC.

**Results:**

The study identified 30 plasma metabolites and 4 urinary metabolites for CRC. Plasma sphingomyelin and urinary lactose, which were positively associated with CRC risk, could be modulated by drug interventions (ie, olipudase alfa, tilactase). Thirteen modifiable risk factors were associated with 9 metabolites, and 8 of these modifiable risk factors were associated with CRC risk. These 9 metabolites mediated the effect of modifiable risk factors (Actinobacteria, body mass index, waist to hip ratio, fasting insulin, smoking initiation) on CRC.

**Conclusion:**

This study identified key metabolite biomarkers associated with CRC and elucidated their mediator roles in the associations between modifiable risk factors and CRC. These findings provide new insights into the etiology and potential therapeutic targets for CRC and the etiological pathways of modifiable environmental factors with CRC.

Colorectal cancer (CRC) is the third-most common malignant tumor and the second-most common cause of cancer death in the world (1). Evidence suggests that metabolic alterations are tied to the occurrence and progression of CRC (2). Some metabolic pathways, such as glycolysis (3), fatty acid metabolism (4), and gut flora metabolism (5), are distinct between colorectal tumors and normal mucosa. A better understanding of these metabolic changes may contribute to uncovering the etiological mechanism of CRC and the development of novel strategies for CRC prevention, diagnosis, and treatment.

Metabolites are the intermediate link between genetic factors or environmental exposures and diseases, and they can accurately reflect the current health state of individuals as the endpoints of cellular pathways and biological processes (6). Currently, alterations in the plasma metabolites have been observed between patients with CRC and controls (7-13). Most of these studies were observational, however, and limited to candidate approaches with few numbers of metabolites, single biological sample sources, or small sample sizes, which restricted their ability to understand the causal role of metabolites in CRC. Furthermore, given that plasma and urine metabolites are strongly influenced by lifestyle factors such as diet, smoking, alcohol, and drugs, these metabolites could be potential mediators underlying the detrimental effect of lifestyle risk factors and hence may represent interventional targets (14).

As an analytic strategy for investigating causal relationships between exposures and outcomes by using genetic variations as instrumental variables, Mendelian randomization has the dual benefits of minimizing confounding and diminishing reverse causality (15). Here, we employed metabolome-wide Mendelian randomization and colocalization analyses to identify causal plasma and urinary metabolites of CRC by integrating human metabolome with genome data from large-scale genome-wide association studies (GWASs). We then evaluated whether the identified CRC-related metabolites can be modulated by pharmacologic or lifestyle interventions.

## Methods

**Figure 1.**
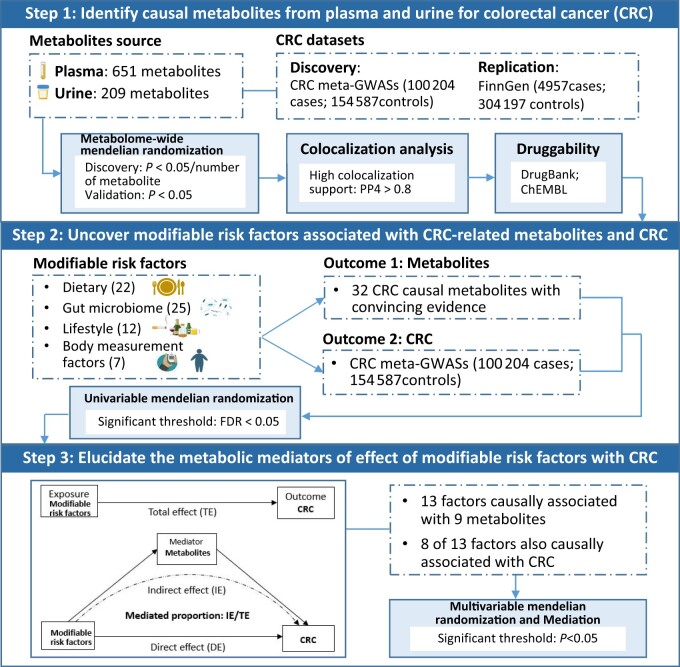
Flowchart of the study design. GWAS = genome-wide association study; PP4 = posterior probability for H4; FDR = false discovery rate.

Figure 1 shows the overall study design. First, we performed a 2-stage (discovery and replication) metabolome-wide Mendelian randomization analysis to examine causal associations of plasma and urinary metabolites with CRC risk, using metabolite quantitative trait locus data derived from 2 large-scale metabolomics GWASs. The statistically significant associations between metabolites and CRC were further prioritized by Bayesian colocalization. Then, we evaluated the druggability of identified metabolites and systematically scanned modifiable risk factors associated with CRC-related metabolites and CRC by employing univariate Mendelian randomization. Last, multivariable Mendelian randomization and mediation analyses were performed to elucidate the metabolic mediators of the associations between modifiable risk factors and CRC.

**Figure 2.**
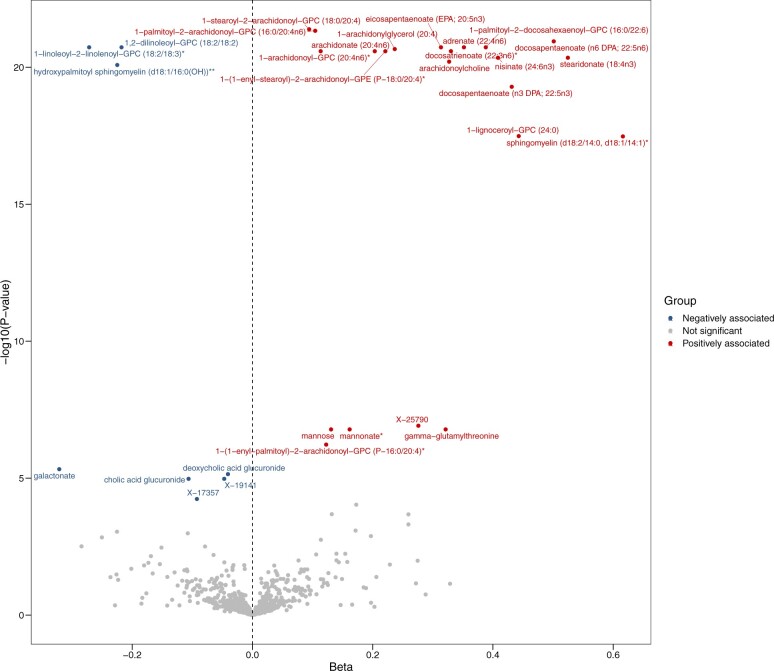
Volcano plot showing results from plasma metabolome-wide Mendelian randomization in the discovery stage. The metabolome-wide Mendelian randomization was performed based on summary statistics of genetic associations of a large-scale plasma metabolomics study and CRC meta-GWAS summary data to test associations of 651 plasma metabolites with CRC risk. Metabolites that survived after Bonferroni correction are labeled. CRC = colorectal cancer.

### Study population and datasets

The largest CRC GWAS dataset (100* *204 cases and 154* *587 controls) to date, covering 22 million single-nucleotide variations (SNVs; formerly single-nucleotide polymorphisms) (16), was employed in the discovery metabolome-wide Mendelian randomization analysis. Details of the study population, genotyping, and imputation have previously been described (16). Independent GWAS summary data covering 20 million SNVs on the basis of 4957 CRC cases and 304* *197 controls from the FinnGen cohort were used in the replication stage (17). Supplementary Table 1 (available online) presents the basic characteristics of these CRC GWASs. Ethics approvals were obtained from the relevant authorities, and all participants provided informed consent (16,17).

Summary statistics of genetic associations with plasma and urinary metabolites were extracted from 2 large-scale metabolomics studies with 690 plasma metabolites (18) and 211 urinary metabolites (19), respectively. These metabolites were measured using an ultra–high-performance liquid chromatography-tandem mass spectrometry platform. Supplementary Table 2 (available online) shows detailed information about metabolomics studies.

Summary statistics of genetic associations for 66 modifiable risk factors (22 dietary factors, 25 gut microbial taxa, 12 lifestyle factors, and 7 obesity-related factors) were extracted from 15 studies. Detailed information about these factors and studies is available in Supplementary Table 3 (available online).

## Statistical analysis

### Metabolome-wide Mendelian randomization analysis

The genetic instruments were selected using metabolite quantitative trait loci from the above-mentioned 2 metabolomics studies. Supplementary Methods (available online) show the details for SNV selection criteria. After matching and harmonizing with CRC outcome data, a total of 1237 instruments for 651 unique plasma metabolites and 233 instruments for 208 unique urinary metabolites remained. Details for all the instrumental variables are shown in Supplementary Tables 4 and 5 (available online).

The *TwoSampleMR* package (20) was used to conduct metabolome-wide Mendelian randomization analysis. The method details are described in Supplementary Methods (available online). A strict Bonferroni correction method was adopted for correction of multiple testing in discovery stage to reduce false-positive findings. For statistically significant metabolites in the discovery dataset, we further conducted Mendelian randomization analysis to replicate their associations using CRC GWAS summary statistics from the FinnGen cohort. Finally, the random-effects meta-analysis was performed to estimate the combined estimate for each metabolite from discovery and replication datasets.

### Bayesian colocalization analysis

Bayesian colocalization analysis was performed using the *coloc* package (21) on the basis of summary statistics of identified metabolites and CRC meta-GWASs to assess whether 2 associated signals (metabolite and CRC risk) were driven by a shared causal genetic variant to distinguish the confounding of linkage disequilibrium (LD). The strong evidence of colocalization was defined as the posterior probability for the hypothesis 4 (H4) greater than 80%. The method details are described in Supplementary Methods (available online).

### Druggability evaluation

We searched the targets and drug information using DrugBank (22) and ChEMBL (23) to evaluate whether the identified metabolites could serve as potential therapeutic targets. DrugBank and ChEMBL prioritized the potential druggable targets by integrating information from text mining, gene function, drug-gene interactions, and expert curation. The information and the development process of drugs that targeted identified metabolites were documented.

### Multivariable Mendelian randomization and mediation analyses

Furthermore, to uncover modifiable risk factors that can modulate CRC-related metabolites, we first employed univariate Mendelian randomization analysis to systematically evaluate the relationships of modifiable risk factors with identified metabolites and CRC risk, respectively. A false discovery rate by Benjamini-Hochberg adjusted *P* < .05 was identified as the significance level. We then performed multivariable Mendelian randomization to test whether metabolites mediated the effect of modifiable factors on CRC. The method details are described in Supplementary Methods (available online). Finally, the mediated proportion was calculated using the formula (total effect ‒ direct effect)/total effect. All statistical analyses were conducted in R, version 4.1.0 (R Foundation for Statistical Computing, Vienna, Austria).

## Results

### Metabolome-wide Mendelian randomization analysis identified 33 metabolites for CRC

The *F* statistics for all instruments of metabolites were above 10, indicating a good strength (Supplementary Tables 4 to 5, available online). Metabolome-wide Mendelian randomization identified that 102 plasma metabolites and 25 urinary metabolites were associated with CRC risk with nominal significance (*P *<* *.05) (Supplementary Tables 6 to 7, available online). Among them, genetically predicted ethylmalonate and methylsuccinate levels in both plasma and urine were positively associated with CRC, while levels of X-19141 and X-12707 in both plasma and urine were inversely associated with CRC (Supplementary Figure 1, available online). After Bonferroni correction, a total of 30 plasma metabolites (*P *<* *7.68 × 10^‒5^) (Table 1, Figure 2) and 4 urinary metabolites (*P *<* *2.39 × 10^‒4^) (Table 1;Supplementary Figure 2, available online) were statistically significantly associated with CRC. Genetically predicted levels of 22 plasma metabolites and 2 urinary metabolites were positively associated with CRC risk, while the other 8 plasma metabolites and 2 urinary metabolites were inversely associated with CRC. For metabolites that had 2 sources and survived after Bonferroni correction in at least 1 source, Mendelian randomization results of both plasma and urine sources and power are shown in Supplementary Table 8 (available online). No evidence of heterogeneity and pleiotropy was observed (*P*_heterogeneity_* *>* *.05, *P*_pleiotropy_* *>* *.05) (Supplementary Table 9, available online). For 6 metabolite pairs, covering a total of 19 metabolites, that had partly overlapping instruments, multivariable Mendelian randomization prioritized 6 metabolites that had a more dominant effect on CRC risk (*P *<* *.008) (Supplementary Table 10, available online). In stratification analysis by ethnicity, most of these metabolite-CRC associations were still statistically significant in White and Asian populations with consistent effect direction (*P *<* *.05) and no statistically significant heterogeneity by ethnicity (Supplementary Table 11, available online).

**Table 1.** Summary results from Mendelian randomization, meta-analysis, and colocalization for metabolome-wide Mendelian randomization identified metabolites

		Mendelian randomization discovery		Mendelian randomization replication		Meta-analysis		Colocalization		
Tissue	Metabolite	Odds ratio	*P*	Odds ratio	*P*	Odds ratio (95% confidence interval)	*P*	PP4 > 80%^a^	Subpathway	Category
Plasma	1-Stearoyl-2-arachidonoyl-GPC (18:0/20:4)	1.10	4.09E-22	1.10	1.05E-03	1.10 (1.08 to 1.12)	1.81E-24	Yes	Phosphatidylcholine	Tier1
Plasma	1-Palmitoyl-2-arachidonoyl-GPC (16:0/20:4n6)	1.11	4.64E-22	1.11	1.74E-03	1.11 (1.09 to 1.13)	3.31E-24	Yes	Phosphatidylcholine	Tier1
Plasma	1,2-Dilinoleoyl-GPC (18:2/18:2)	0.80	1.86E-21	0.81	1.62E-03	0.80 (0.77 to 0.84)	1.23E-23	Yes	Phosphatidylcholine	Tier1
Plasma	1-Linoleoyl-2-linolenoyl-GPC (18:2/18:3)*	0.76	1.86E-21	0.77	1.62E-03	0.76 (0.72 to 0.80)	1.23E-23	Yes	Phosphatidylcholine	Tier1
Plasma	1-Palmitoyl-2-docosahexaenoyl-GPC (16:0/22:6)	1.47	1.86E-21	1.46	1.62E-03	1.47 (1.37 to 1.59)	1.23E-23	Yes	Phosphatidylcholine	Tier1
Plasma	Adrenate (22:4n6)	1.42	1.86E-21	1.41	1.62E-03	1.42 (1.33 to 1.52)	1.23E-23	Yes	Long chain polyunsaturated fatty acid	Tier1
Plasma	EPA (20:5n3)	1.37	1.86E-21	1.36	1.62E-03	1.37 (1.29 to 1.45)	1.23E-23	Yes	Long chain polyunsaturated fatty acid	Tier1
Plasma	1-Arachidonylglycerol (20:4)	1.27	2.16E-21	1.27	1.09E-03	1.27 (1.21 to 1.33)	9.87E-24	Yes	Monoacylglycerol	Tier1
Plasma	Docosatrienoate (22:3n6)*	1.39	2.58E-21	1.36	2.71E-03	1.39 (1.30 to 1.48)	2.81E-23	Yes	Long chain polyunsaturated fatty acid	Tier1
Plasma	1-(1-Enyl-stearoyl)-2-arachidonoyl-GPE (P-18:0/20:4)*	1.25	2.59E-21	1.24	1.79E-03	1.25 (1.19 to 1.30)	1.88E-23	Yes	Plasmalogen	Tier1
Plasma	1-Arachidonoyl-GPC (20:4n6)*	1.12	2.59E-21	1.12	1.79E-03	1.12 (1.10 to 1.15)	1.87E-23	Yes	Lysophospholipid	Tier1
Plasma	Arachidonate (20:4n6)	1.23	2.59E-21	1.22	1.79E-03	1.23 (1.18 to 1.27)	1.88E-23	Yes	Long chain polyunsaturated fatty acid	Tier1
Plasma	Nisinate (24:6n3)	1.50	4.48E-21	1.47	2.60E-03	1.50 (1.38 to 1.63)	4.65E-23	Yes	Long chain polyunsaturated fatty acid	Tier1
Plasma	Stearidonate (18:4n3)	1.69	4.48E-21	1.64	2.60E-03	1.69 (1.52 to 1.87)	4.65E-23	Yes	Long chain polyunsaturated fatty acid	Tier1
Plasma	Arachidonoylcholine	1.39	6.23E-21	1.39	1.63E-03	1.39 (1.30 to 1.48)	4.14E-23	Yes	Fatty acid metabolism (acyl choline)	Tier1
Plasma	Hydroxypalmitoyl sphingomyelin (d18:1/16:0(OH))**	0.80	8.23E-21	0.80	1.87E-03	0.80 (0.76 to 0.84)	6.20E-23	Yes	Sphingomyelins	Tier1
Plasma	Docosapentaenoate (n3 DPA; 22:5n3)	1.54	5.14E-20	1.39	1.87E-02	1.52 (1.40 to 1.66)	4.08E-21	Yes	Long chain polyunsaturated fatty acid	Tier1
Plasma	1-Lignoceroyl-GPC (24:0)	1.56	3.25E-18	1.65	7.58E-04	1.57 (1.43 to 1.72)	1.12E-20	Yes	Lysophospholipid	Tier1
Plasma	γ-Glutamylthreonine	1.38	1.66E-07	1.74	4.57E-03	1.44 (1.20 to 1.72)	6.18E-05	Yes	γ-Glutamyl amino acid	Tier1
Plasma	Mannonate*	1.18	1.66E-07	1.32	4.57E-03	1.20 (1.10 to 1.31)	6.18E-05	Yes	Food component/plant	Tier1
Plasma	Mannose	1.14	1.66E-07	1.25	4.57E-03	1.16 (1.08 to 1.25)	6.17E-05	Yes	Fructose, mannose, and galactose metabolism	Tier1
Plasma	Docosapentaenoate (n6 DPA; 22:5n6)	1.65	1.13E-21	1.28	8.37E-02	1.51 (1.19 to 1.91)	6.83E-04	Yes	Long chain polyunsaturated fatty acid	Tier2
Plasma	Sphingomyelin (d18:2/14:0, d18:1/14:1)*	1.85	3.34E-18	1.97	1.01E-03	1.86 (1.63 to 2.13)	1.47E-20	No	Sphingomyelins	Tier2
Plasma	1-(1-Enyl-palmitoyl)-2-arachidonoyl-GPC (P-16:0/20:4)*	1.13	5.96E-07	1.09	3.14E-01	1.13 (1.08 to 1.18)	3.77E-07	Yes	Plasmalogen	Tier2
Plasma	Galactonate	0.73	4.71E-06	0.62	5.44E-03	0.71 (0.62 to 0.81)	1.22E-07	No	Fructose, mannose, and galactose metabolism	Tier2
Plasma	Deoxycholic acid glucuronide	0.96	7.14E-06	0.95	1.59E-02	0.96 (0.94 to 0.97)	4.17E-07	No	Secondary bile acid metabolism	Tier2
Plasma	Cholic acid glucuronide	0.90	1.06E-05	0.87	1.96E-02	0.89 (0.86 to 0.94)	7.15E-07	No	Primary bile acid metabolism	Tier2
Plasma	X-19141	0.95	1.06E-05	0.94	1.95E-02	0.95 (0.93 to 0.97)	7.08E-07	No	Unknown	Tier2
Plasma	X-25790	1.32	1.22E-07	1.17	3.54E-01	1.30 (1.18 to 1.44)	9.81E-08	No	Unknown	Tier3
Plasma	X-17357	0.91	5.77E-05	1.02	7.66E-01	0.95 (0.85 to 1.05)	3.25E-01	No	Unknown	Tier3
Urine	Lactose	1.14	4.40E-05	1.27	1.31E-03	1.18 (1.06 to 1.30)	1.50E-03	Yes	Disaccharides and oligosaccharides	Tier1
Urine	X-19141	0.94	9.97E-06	0.92	1.90E-02	0.94 (0.91 to 0.96)	6.56E-07	No	Unknown	Tier2
Urine	X-19434 retired for cholic acid glucuronide	0.96	1.12E-05	0.95	3.20E-02	0.95 (0.94 to 0.97)	1.09E-06	No	Unknown	Tier2
Urine	11-Ketoetiocholanolone sulfate	1.06	1.16E-05	1.08	1.94E-02	1.06 (1.04 to 1.09)	7.77E-07	No	Androgenic steroids	Tier2

a PP4 values were all higher than 80% under different windows (±250 kilobase, kb or ±500 kb). PP4 = posterior probability for H4; E = exponent.

In the replication stage, 27 plasma metabolites and 4 urinary metabolites were successfully validated in the FinnGen dataset (*P *<* *.05) (Table 1). In the meta-analysis of discovery and replication datasets, 29 plasma metabolites and 4 urinary metabolites displayed statistically significant associations with CRC risk, which could be classified into 13 subcategories (Table 1).

### 24 Metabolites were supported by colocalization evidence

A total of 24 metabolites (23 plasma and 1 urinary) were supported by strong evidence of colocalization, with posterior probability for H4 greater than 80% across different window sizes, suggesting high probability for a shared causal genetic variant for metabolites and CRC risk (Table 1; Supplementary Table 12, available online). Combining the above evidence, CRC-related metabolites were classified into 3 tiers: 22 metabolites passed all tests and were classified into tier 1, 10 metabolites failed in Mendelian randomization replication or colocalization and were classified into tier 2, and 2 metabolites failed in both replication Mendelian randomization and colocalization and were classified into tier 3.

### Two metabolites as therapeutic targets by drugs

Druggability evaluation showed that 2 of the CRC-related metabolites (sphingomyelin [d18:2/14:0, d18:1/14:1]*, lactose) have been targeted by pharmacologic intervention (Supplementary Table 13, available online). A drug (olipudase alfa) targeting sphingomyelin (d18:2/14:0, d18:1/14:1)* has been used to treat acid sphingomyelinase deficiency, acting to catalyze the hydrolysis of sphingomyelin to reduce the amount of sphingomyelin. A drug (tilactase) targeting lactose has been used to treat lactose intolerance and developed to treat irritable bowel syndrome.

### Nine metabolites as interventional targets by modifiable factors

**Figure 3.**
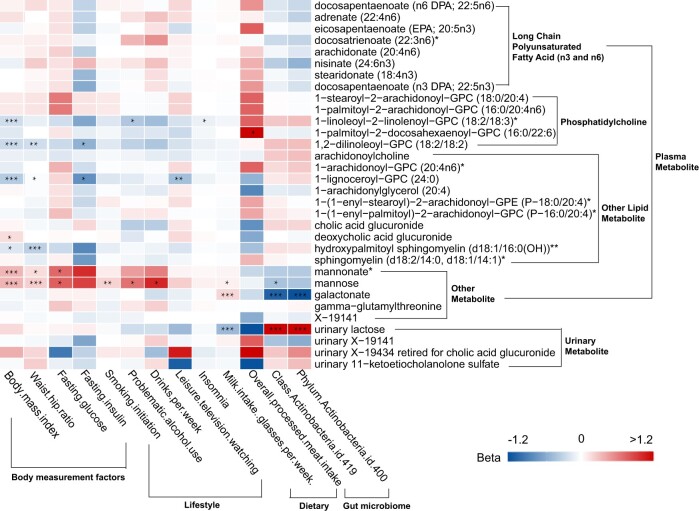
Heatmap showing results from Mendelian randomization of modifiable risk factors with colorectal cancer–related metabolites. * False discovery rate corrected *P* < .05. ** False discovery rate corrected *P* < .01. *** False discovery rate corrected *P* < .001.

In univariable Mendelian randomization analysis of modifiable factors with 32 metabolites that have convincing evidence (tiers 1 and 2), we found that a total of 13 modifiable risk factors (2 dietary factors, 2 gut microbial taxa, 5 lifestyle factors, 4 obesity-related factors) were associated with 9 CRC-related metabolites (false discovery rate < 0.05) (Figure 3; Supplementary Table 14, available online), and 8 of 13 modifiable factors were also associated with CRC risk (false discovery rate < 0.05) (Supplementary Table 15, available online).

### Metabolites partially mediate the effect of modifiable factors on CRC

**Figure 4.**
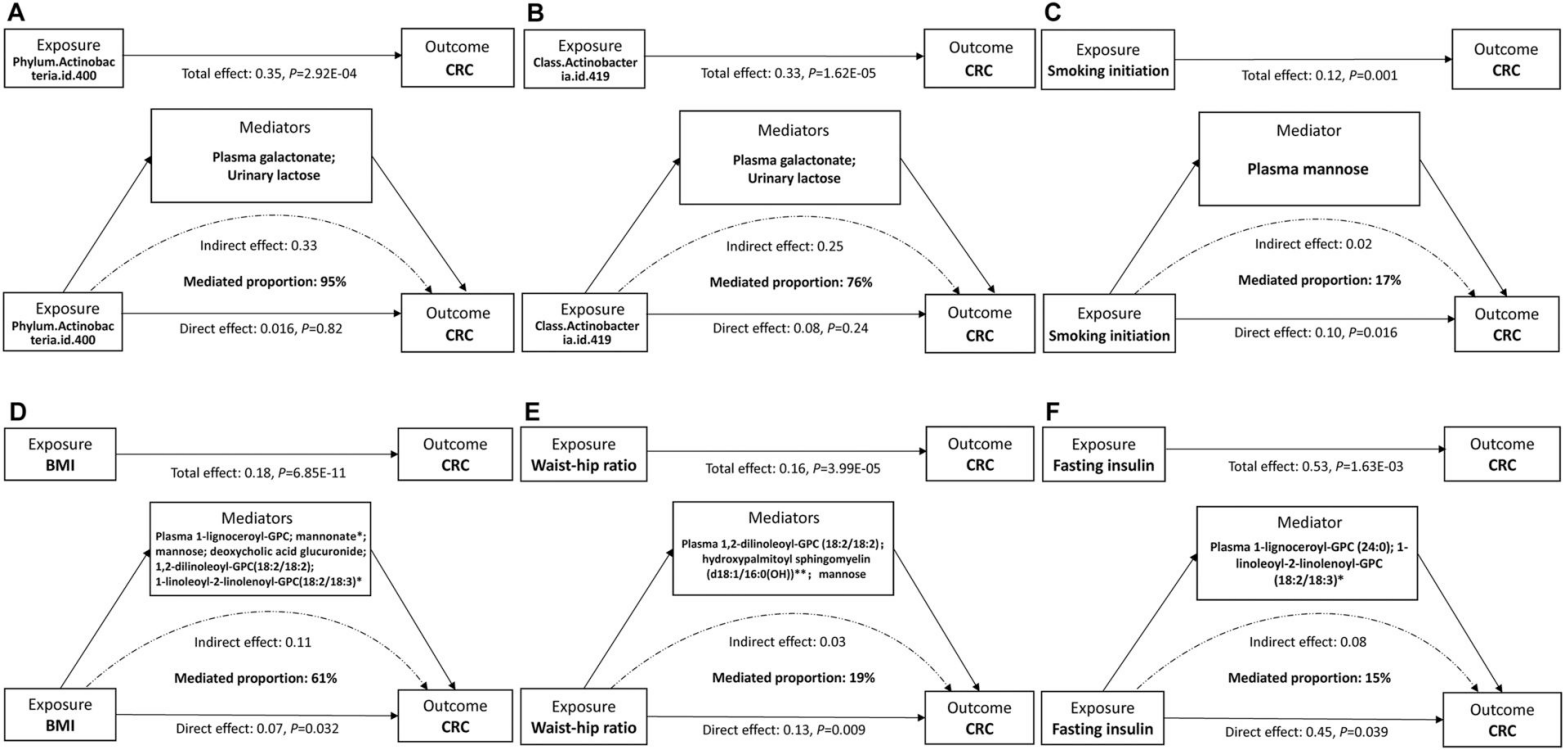
Metabolic mediators of the relationships between modifiable risk factors and CRC. **A**) Phylum Actinobacteria effect on CRC mediated by plasma galactonate and urinary lactose. **B**) Class Actinobacteria effect on CRC mediated by plasma galactonate and urinary lactose. **C**) Smoking initiation effect on CRC mediated by plasma 1-lignoceroyl-GPC (24:0), 1-linoleoyl-2-linolenoyl-GPC (18:2/18:3)*. **D**) BMI effect on CRC mediated by plasma 1-lignoceroyl-GPC, mannonate*, mannose, deoxycholic acid glucuronide, 1,2-dilinoleoyl-GPC(18:2/18:2), and 1-linoleoyl-2-linolenoyl-GPC(18:2/18:3)*. **E**) Waist to hip ratio effect on CRC mediated by plasma 1,2-dilinoleoyl-GPC (18:2/18:2), hydroxypalmitoyl sphingomyelin (d18:1/16:0(OH))**, and mannose. **F**) Fasting insulin effect on CRC mediated by plasma mannose. BMI = body mass index; CRC = colorectal cancer.

Figure 4 shows the modifiable factor-metabolites-CRC pairs with mediating effects. For 8 modifiable factor-metabolites-CRC pairs, 7 pairs (except for milk intake) had full summary data and were further evaluated using multivariable Mendelian randomization. The associations of phylum Actinobacteria, class Actinobacteria, body mass index (BMI), waist to hip ratio, fasting insulin, and smoking initiation with CRC risk were attenuated in the multivariable Mendelian randomization analyses, with adjustment for metabolites (Figure 4), whereas the association of leisure television watching with CRC became stronger (Supplementary Figure 3, available online). Among them, genetically predicted levels of plasma galactonate and urinary lactose mediated 95% of the effect of phylum Actinobacteria on CRC and 76% of the effect of class Actinobacteria on CRC (Figure 4).

## Discussion

In this study, we performed a comprehensive investigation on associations of plasma and urinary metabolites with CRC risk. The discovery metabolome-wide Mendelian randomization analysis identified 30 plasma and 4 urinary metabolites associated with CRC, and most of them showed cross-ethnicity effect consistencies. The replication Mendelian randomization validated 30 candidate metabolites, and 24 metabolites were supported by colocalization evidence. Collectively, 22, 10, and 2 metabolites were classified into the most convincing evidence (tier 1), convincing evidence (tier 2), and low evidence (tier 3) groups, respectively. Druggability evaluation prioritized 2 CRC-related metabolites (ie, sphingomyelin, lactose) that could be modified by drug interventions; additionally, 9 of these metabolites could be modulated by modifiable risk factors. Multivariable Mendelian randomization analyses indicated that the effect of modifiable factors (ie, Actinobacteria, smoking initiation, BMI, waist to hip ratio, and fasting insulin) on CRC were partially mediated by these identified metabolites.

Our findings indicated a potentially important role of long chain polyunsaturated fatty acid (n3 and n6) (adrenate [22:4n6], arachidonate [20:4n6], stearidonate [18:4n3], EPA, n3 DPA, n6 DPA, nisinate [24:6n3], docosatrienoate [22:3n6]*) in CRC liability. The observed relationships of adrenate (22:4n6), arachidonate (20:4n6), and stearidonate (18:4n3) with CRC are consistent with previous findings (24-26), but current evidence of EPA and DPA on CRC are conflicting. Previous Mendelian randomization studies and our findings indicated that high plasma EPA, n3 DPA, and n6 DPA were positively associated with CRC (25,27). Observational studies showed null association between blood DPA and EPA levels and CRC and even inverse association between dietary their intake and CRC risk (27). Given that in dietary assessments it is difficult to be precise and observational studies are susceptible to confounding factors, further intervention studies are needed to explain these inconsistent findings. We additionally found positive associations of nisinate (24:6n3) and docosatrienoate (22:3n6)* with CRC. In rodents, nisinate (24:6n3) is both a product of and a precursor to docosahexaenoic acid in the n-3 PUFA biosynthetic pathway, and docosahexaenoic acid has been reported to influence the invasion in CRC cells (28).

Our findings also suggested a potential role of phosphatidylcholine and choline metabolites in CRC development. Phosphatidylcholine is a structural component of mammalian membranes and an important source of lipid second messengers; it has been reported to be closely involved in carcinogenesis (29). Choline metabolites are derived from the synthesis and catabolism of phosphatidylcholine, and the phosphatidylcholine cycle of synthesis and catabolism helps maintain the proliferative phenotype of tumor cells and supports tumor progression and culminate in resistance to therapy (30). Consistently, we found a detrimental effect of plasma arachidonoylcholine (an acylated derivative of choline) on CRC. We found that genetically predicted higher levels of plasma 1-stearoyl-2-arachidonoyl-GPC (18:0/20:4), 1-palmitoyl-2-arachidonoyl-GPC (16:0/20:4n6), and 1-palmitoyl-2-docosahexaenoyl-GPC (16:0/22:6) and lower levels of 1,2-dilinoleoyl-GPC (18:2/18:2) and 1-linoleoyl-2-linolenoyl-GPC (18:2/18:3)* were associated with an increased risk of CRC, which expanded the causal evidence of PC subclass metabolites with CRC.

We observed detrimental effects of plasma mannose and urinary lactose and the protective effect of plasma galactonate on CRC. Consistently, Long et al. (9) found that patients with CRC had higher levels of mannose than controls. A randomized controlled trial indicated that intake of lactose-rich foods increased the risk of diarrhea in patients with CRC (31). Galactonate is a metabolic breakdown product of galactose, a monosaccharide that together with glucose forms lactose. Intestinal galactose shows a protective effect against colon cancer through binding lectins and inhibiting mucosal proliferation, and the lower level of galactose leads to the pathogenetic process of CRC (32). γ-Glutamylthreonine is a dipeptide composed of γ-glutamate and threonine. Both our study and a previous Mendelian randomization study reported a positive association between blood γ-glutamylthreonine and CRC (12).

The identified metabolites could be modulated by either pharmacological intervention or modifiable factors. Specifically, 2 metabolites (sphingomyelin, lactose) could be modulated by drugs used to treat acid sphingomyelinase deficiency or irritable bowel syndrome. Also, avoiding excessive consumption of food products containing lactose could be efficient. Nine of the identified CRC-related metabolites were observed to be affected by modifiable factors. The identified metabolites partially mediated the effect of Actinobacteria, BMI, waist to hip ratio, fasting insulin, and smoking initiation on CRC. The positive association between Actinobacteria and CRC was found to be mediated by higher levels of urinary lactose and decreased levels of plasma galactonate. Similarly, a nested case-control study reported that compared with controls, patients with CRC had more abundant oral Actinobacteria (33). Other studies also showed that Actinobacteriota was 1 of the dominant colonic mucosal microbiota in patients with CRC (34) and has shown abundance even in the colorectal adenomas stage (35). Because these results are derived by retrospective studies, however, they may be due to reverse causality. The association of Actinobacteria with lactase gene (*LCT*) has previously been documented, suggesting an interaction of Actinobacteria with the gut and lactose metabolism (36). Consumption of lactose may increase its availability in colonic bacteria (such as Actinobacteria and Negativibacillus) that use it as the energy source for which to compete, especially for adults with lactase deficiency (36). An abundance of Actinobacteria may lead to metabolic disorders of lactose and galactonate and could contribute to an increased CRC risk. Plasma mannose was found to partially mediate the relationships of smoking initiation, BMI, and waist to hip ratio with CRC. Similarly, Long et al. (9) found a statistically significant joint effect of smoking with mannose and a statistically significant interaction between BMI and mannose in modifying CRC risk. We found that fasting insulin was positively associated with CRC and that the association was partially mediated by plasma 1-lignoceroyl-GPC (24:0) and 1-linoleoyl-2-linolenoyl-GPC (18:2/18:3)*. Gut microbiota, obesity, insulin resistance, and smoking have been linked to the etiology of CRC by abundant evidence from observational studies (37). We expanded the causal evidence and elucidated the potential etiologic metabolic pathways of these modifiable factors with CRC.

The current study has several strengths. First, to our knowledge, this study is the first to comprehensively evaluate the causal associations of metabolites from plasma and urine with CRC, which helps provide new insights into the etiology and potential therapeutic targets for CRC. Second, the present study was performed by employing Mendelian randomization design and colocalization based on well-designed GWASs with large sample sizes, which enhanced the statistical power and reduced the risk of confounding bias and reverse causation. Additionally, we assessed the mediating role of CRC-related metabolites in modifiable factors and CRC, which provided new insights into the etiologic pathways of modifiable environmental factors with CRC. Several limitations of this study should be acknowledged, as well. First, the strict significance threshold of Bonferroni correction in discovery Mendelian randomization may filter out some important metabolites, although these findings may be less prone to false-positive errors. Second, instrumental variables for gut microbiota in multivariable Mendelian randomization stage were selected at a more lenient threshold value (*P *<* *5 × 10^−6^), as indicated by the original microbiome GWAS of MiBioGen consortium and other Mendelian randomization studies that the selection of associated SNVs using lenient *P* value thresholds had the greatest explained variance on microbial features (38,39). The *F* statistics of all instrumental variables used in the current study were above 10, indicating low risk of weak instruments bias. Third, participants of urine metabolites GWASs had reduced kidney function, which may not be representative of the general population, despite the relevant genetic effects on most urine metabolite concentrations between individuals with reduced kidney function and the general population (19). Further studies based on the general population are required. Fourth, the mediation effects of modifiable factor-metabolite-CRC pairs were discovered mainly by statistical analysis. Further experiment research is needed to verify these findings and elucidate the underlying biological mechanism.

This study identified key metabolites with a potential causal association with CRC risk and elucidated the metabolic mediators of the effect of modifiable risk factors on CRC. Our findings provide new insights into the etiology and potential therapeutic targets for CRC and the etiologic pathways of modifiable environmental factors with CRC. Further interventional studies are needed to evaluate whether the concentrations of these metabolites could be modified through drug intervention or lifestyle changes and ultimately reduce CRC risk.

## Supplementary Material

djae089_Supplementary_Data

## Data Availability

The results of this study are included in this published article and its supplementary information files. The GWAS summary data of CRC meta-GWAS are available through the GWAS catalog (accession No. GCST90129505). The GWAS summary data of FinnGen are available at https://www.finngen.fi/en/access_results. Further information is available from the corresponding author upon request.
