# Supplementary material for: Systematic investigation of genetically determined plasma and urinary metabolites to discover potential interventional targets for colorectal cancer
Source: J Natl Cancer Inst. 2024 Apr 22;116(8):1303–12. doi: 10.1093/jnci/djae089 (PMC11308169; doi:10.1093/jnci/djae089)
Supplement: djae089_Supplementary_Data [file djae089_supplementary_data.zip › djae089_Supplementary_Data/JNCI-24-0023R1-Supplementary Methods and Figures-clean.docx]

**Supplementary Methods and Figures for**

**Systematic investigation of genetically determined plasma and** **urinary metabolites to discover potential** **interventional targets for colorectal cancer**

**Supplementary Methods**

**Metabolome-****wide** **Mendelian randomization analysis**

The genetic instruments were selected using mQTLs from two metabolomic studies based on the following criteria: (i) SNPs associated with any metabolite (*P*<5x10^-8^) were selected; (ii) For each metabolite, SNPs were selected by linkage disequilibrium (LD) clumping to identify independent mQTLs (r^2^<0.001). The R^2^ and F-statistic also were calculated to evaluate the strength of genetic instruments. After matching and harmonizing with CRC outcome data, a total of 1,237 instruments for 651 unique plasma metabolites and 233 instruments for 208 unique urinary metabolites remained. Details for all the instrumental variables (IVs) are shown in **Supplementary Tables 4-5**.

The “TwoSampleMR” package [1] was used to conduct metabolome-wide MR analysis. When metabolites had only one instrument, the Wald ratio method was employed to evaluate the log odds change in CRC risk for per standard deviation (SD) increment of metabolite level as proxied by the genetic instrument. When metabolites had two or more genetic instruments, the inverse-variance weighted (IVW) method was used to estimate the MR effects. Overall, the metabolite levels were proxied by the genetic instruments, and the estimated MR effects were described as the effects of genetically determined or genetically predicted metabolite levels on CRC risk. The heterogeneity of the IVs was evaluated based on the Q statistic by heterogeneity test. Additional models including simple mode, weighted median, weighted mode, and MR-Egger also were used to account for potentially horizontal pleiotropy [2]. A strict Bonferroni correction method was adopted for correction of multiple testing in discovery stage to reduce false-positive findings. For statistically significant metabolite biomarkers in the discovery dataset, we further conducted MR analysis to replicate their associations using CRC GWAS summary statistics from the FinnGen cohort. Finally, the random-effects meta-analysis was performed to estimate the combined estimate for each metabolite from discovery and replication datasets.

For initially identified CRC-related metabolites with partly overlapping instruments, we additionally performed multivariable MR (MVMR) [3] to determine the direct impact of each metabolite on CRC risk while accounting for the effects of other metabolites. MVMR has advantages in the effective management of the complexities arising from interdependences among genetic variants linked to diverse exposures by including multiple exposures that interact. Since MVMR requires sufficient numbers of instruments (more than the number of exposures) to estimate effects and standard errors, we selected instruments under a more lenient clumping threshold value (LD r^2^ < 0.1 to 0.2) in MVMR analysis for metabolite pairs that had insufficient instruments (less than the number of metabolites) under LD r^2^ < 0.001. A p-value of < 0.008 (0.05/number of tests) was identified as the significance level.

To test whether the initially identified associations of metabolites with CRC remained significant in only white or Asian populations, we performed MR analysis of identified metabolites with CRC risk in by employing CRC GWAS summary data from white (78,473 cases and 107,143 controls) [4] and Asian populations (22,775 cases and 47,731 controls) (**Supplementary Table 1**) [5], respectively. Heterogeneity across ethnic groups was also tested to evaluate whether the metabolite-CRC association differed between white and Asians. The mRnd method was used to calculate statistical power [6]. R software 4.1.0 was used to conduct these analyses.

**Bayesian** **colocalization analysis**

Bayesian colocalization analysis was further performed, using the “coloc” package [7] based on summary statistics of identified metabolite biomarkers and CRC meta-GWASs, to assess whether two associated signals (metabolite and CRC risk) were driven by a shared causal genetic variant to distinguish the confounding of LD. The colocalization analysis was conducted under five hypotheses: (i) H0, there was no causal genetic variant for both phenotypes (metabolite and CRC) in the genomic locus; (ii) and (iii) there was one causal variant for metabolite only (H1) or CRC only (H2), respectively; (iv) H3, there were two distinct causal variants for metabolite and CRC; (v) H4, there was a shared causal genetic variant for metabolite and CRC. We used default parameters and prior definitions. For each metabolite, SNPs within ±500 kb of the mQTL were included. For metabolites with more than one mQTL, colocalization analysis was performed based on each mQTL, respectively, and the mQTL with the largest posterior probability for H4 (PP4) was reported. Given that colocalization may be sensitive to window sizes, additional analysis based on a window size of ±250 kb was performed to assess the robustness of the results. Strong evidence of colocalization was defined as the PP4 of > 80% under different windows.

Metabolites that passed all tests (discovery MR, replication MR, colocalization) were classified into tier 1 (the most convincing evidence group). Metabolites that failed in MR replication or colocalization were classified into tier 2 (convincing evidence group). Metabolites that failed in both replication MR and colocalization were classified into tier 3 (low evidence group).

**Druggability evaluation**

We searched the targets and drug information using DrugBank [8] and ChEMBL [9] databases to evaluate whether the identified metabolite biomarkers could serve as potential therapeutic targets. DrugBank and ChEMBL prioritized the potential druggable targets by integrating information from text mining, gene function, drug-gene interactions, and expert curation. The information and the development process of drugs that targeted identified metabolites were documented.

**Multivariable MR and mediation analyses**

Furthermore, to uncover modifiable risk factors (dietary: macronutrients and food groups; gut microbiome; lifestyle: smoking, drinking, sleep, and physical activity related factors; and obesity-related factors) that can modulate the target metabolites related to CRC, we firstly employed univariate MR analysis to systematically evaluate the relationships of modifiable risk factors with identified metabolite biomarkers and CRC risk. A false discovery rate (FDR) by Benjamini-Hochberg adjusted p-value of < 0.05 was identified as the significance level. We then performed MVMR to test whether metabolites mediated the effect of modifiable risk factors on CRC. MVMR requires a sufficient number of instrumental variables (IVs), while gut microbiome (*class.Actinobacteria*, *phylum.Actinobacteria*) had only one IV at *P*<5×10^-8^ level. Considering that IVs with *P* values no more than 1×10^-5^ level can provide the largest explained variance on microbial features [10], meanwhile minimizing the concerns of false positive genetic variants, we used a more lenient threshold value (*P*<5x10^-6^) on the selection of genetic instruments for the gut microbiome in MVMR stage. Finally, the mediated proportion was calculated by the formula: (total effect - direct effect) / total effect. The total effect of modifiable factors on CRC was derived from univariable MR of modifiable factors with CRC, and the direct effect was derived from multivariable MR, controlling for the mediator (metabolites). All statistical analyses were conducted in R Software 4.1.0.

**References**

1. Hemani G, Zheng J, Elsworth B, Wade KH, Haberland V, Baird D *et al*: The MR-Base platform supports systematic causal inference across the human phenome. eLife 2018; 7.

2. Bowden J, Davey Smith G, Haycock PC, Burgess S: Consistent Estimation in Mendelian Randomization with Some Invalid Instruments Using a Weighted Median Estimator. Genetic epidemiology 2016; 40(4):304-314.

3. Burgess S, Thompson SG: Multivariable Mendelian randomization: the use of pleiotropic genetic variants to estimate causal effects. American journal of epidemiology 2015; 181(4):251-260.

4. Fernandez-Rozadilla C, Timofeeva M, Chen Z, Law P, Thomas M, Schmit S *et al*: Deciphering colorectal cancer genetics through multi-omic analysis of 100,204 cases and 154,587 controls of European and east Asian ancestries. Nature genetics 2023; 55(1):89-99.

5. Lu Y, Kweon SS, Tanikawa C, Jia WH, Xiang YB, Cai Q *et al*: Large-Scale Genome-Wide Association Study of East Asians Identifies Loci Associated With Risk for Colorectal Cancer. Gastroenterology 2019; 156(5):1455-1466.

6. Brion MJ, Shakhbazov K, Visscher PM: Calculating statistical power in Mendelian randomization studies. International journal of epidemiology 2013; 42(5):1497-1501.

7. Giambartolomei C, Vukcevic D, Schadt EE, Franke L, Hingorani AD, Wallace C *et al*: Bayesian test for colocalisation between pairs of genetic association studies using summary statistics. PLoS genetics 2014; 10(5):e1004383.

8. Wishart DS, Feunang YD, Guo AC, Lo EJ, Marcu A, Grant JR *et al*: DrugBank 5.0: a major update to the DrugBank database for 2018. Nucleic acids research 2018; 46(D1):D1074-d1082.

9. Mendez D, Gaulton A, Bento AP, Chambers J, De Veij M, Félix E *et al*: ChEMBL: towards direct deposition of bioassay data. Nucleic acids research 2019; 47(D1):D930-d940.

10. Sanna S, van Zuydam NR, Mahajan A, Kurilshikov A, Vich Vila A, Võsa U *et al*: Causal relationships among the gut microbiome, short-chain fatty acids and metabolic diseases. Nature genetics 2019; 51(4):600-605.

**Supplementary Figures**

**Supplementary Figure 1** Venn Diagram showing results from plasma and urinary metabolome-wide Mendelian randomization (MR). Numbers represent the amounts of metabolites with *P*<0.05.

**Supplementary Figure 2** Volcano plot showing results from urinary metabolome-wide Mendelian randomization (MR).

**Supplementary Figure 3** Metabolic mediator of the relationship between leisure television watching and colorectal cancer (CRC).


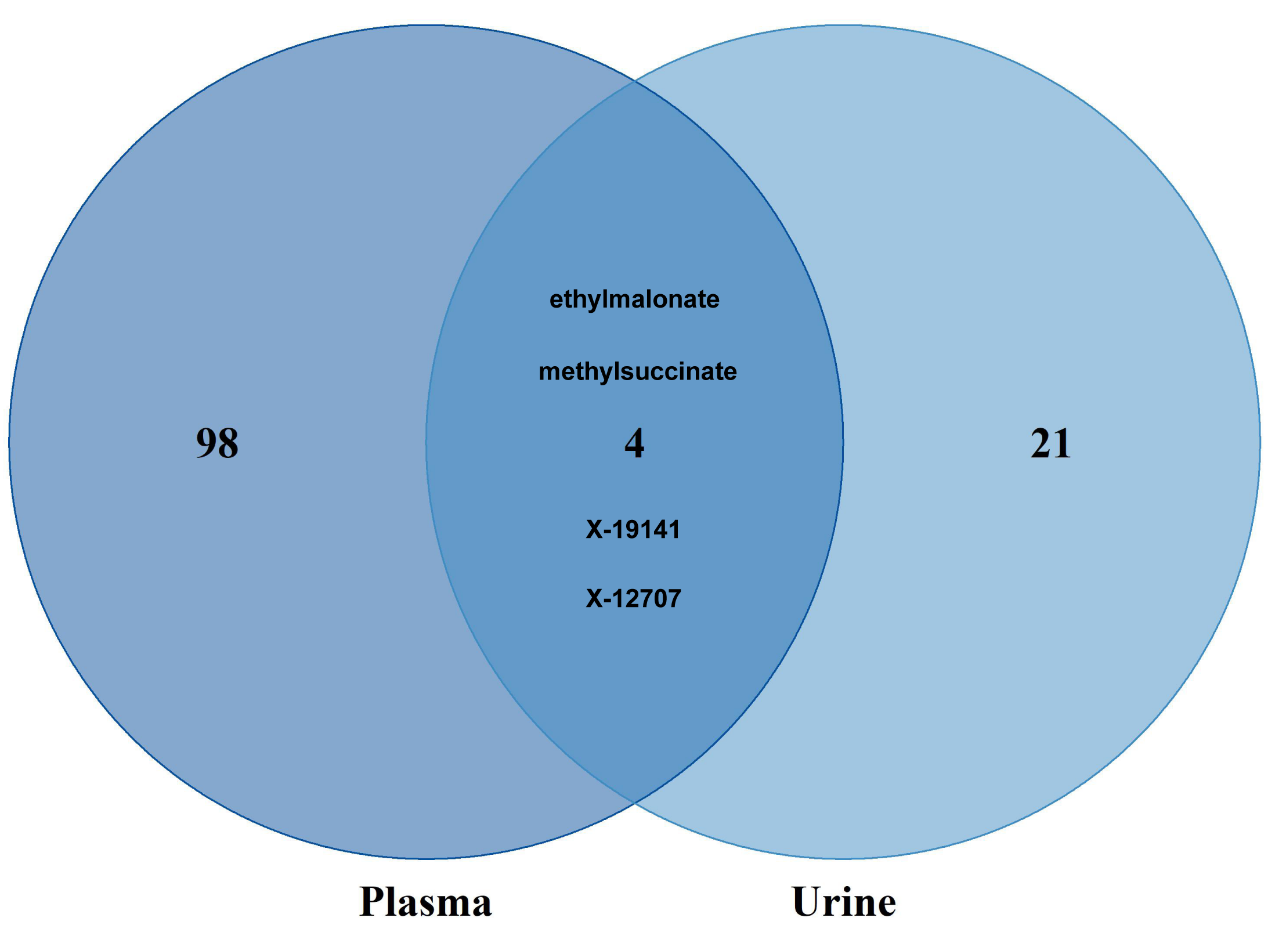


**Supplementary Figure 1** Venn Diagram showing results from plasma and urinary metabolome-wide Mendelian randomization (MR). Numbers represent the amounts of metabolites with *P*<0.05.


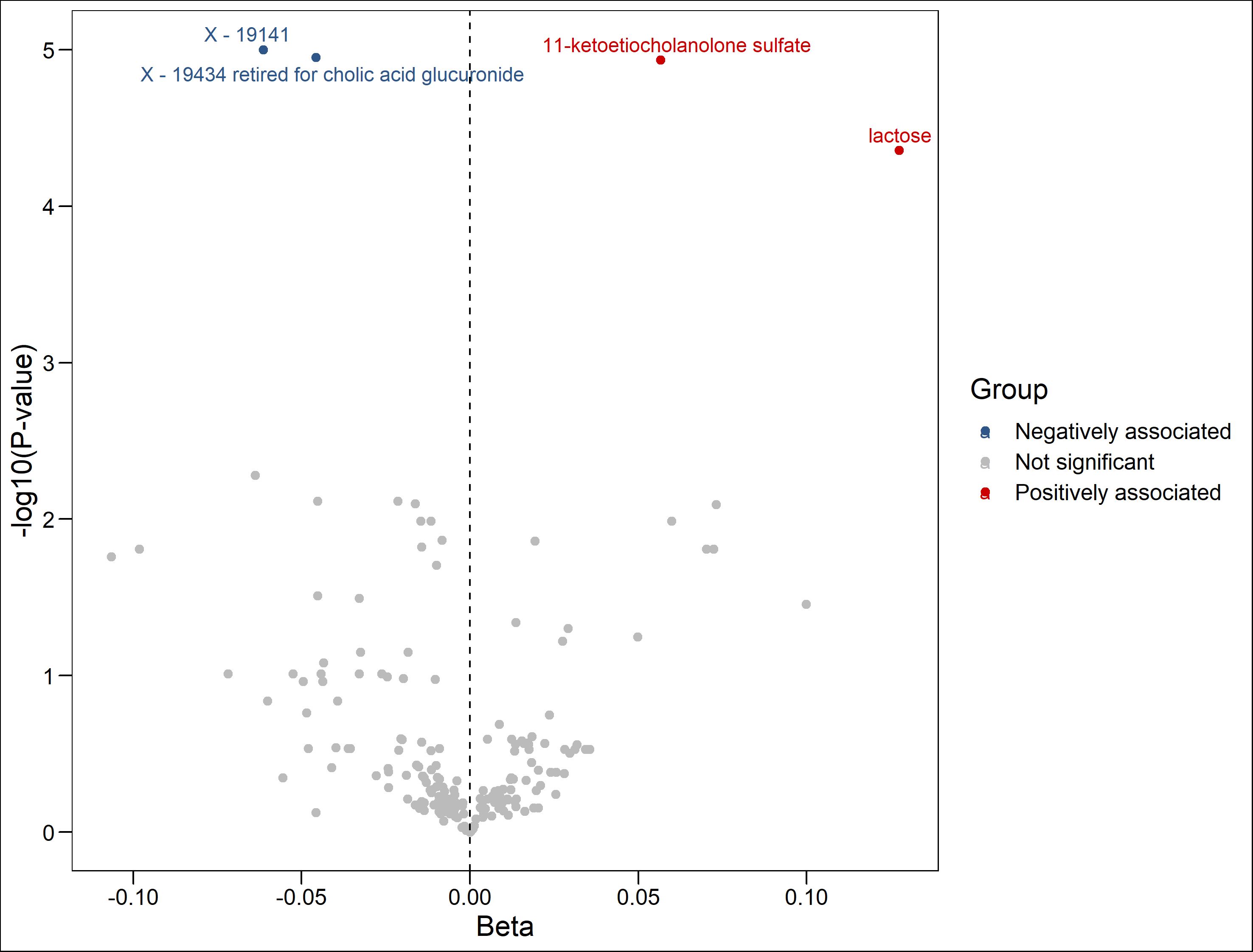


**Supplementary Figure 2** Volcano plot showing results from urinary metabolome-wide Mendelian randomization (MR).


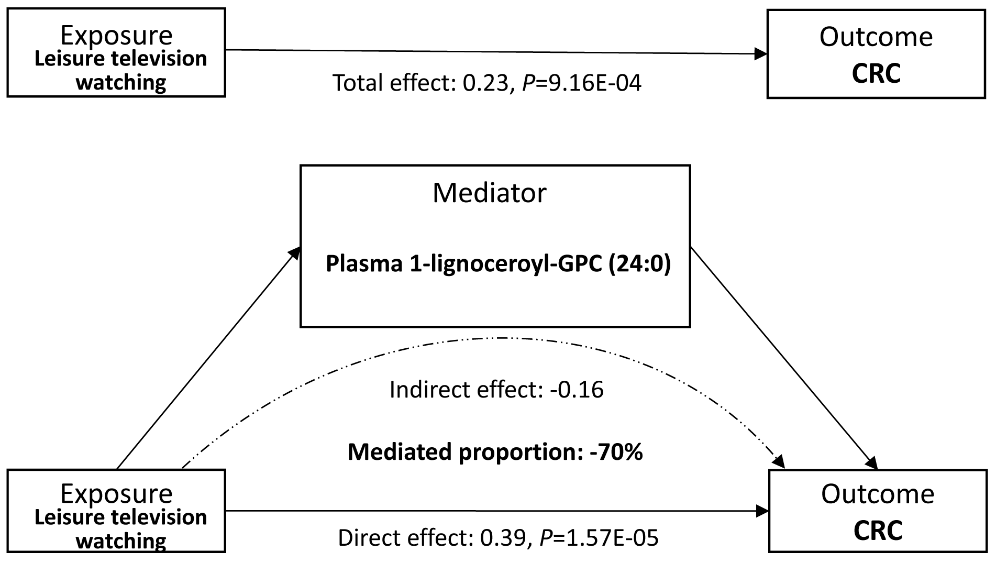


**Supplementary Figure 3** Metabolic mediator of the relationship between leisure television watching and colorectal cancer (CRC).
